# Validation of the Italian version of the Parkinson’s Disease- Cognitive Functional Rating Scale

**DOI:** 10.1007/s00702-024-02746-6

**Published:** 2024-01-27

**Authors:** Michela Garon, Luca Weis, Antònia Siquier, Eleonora Fiorenzato, Francesca Pistonesi, Valeria Cianci, Margherita Canesi, Francesca Pesce, Elisa Reali, Beatrice Pozzi, Ioannis Ugo Isaias, Chiara Siri, Gabriella Santangelo, Sofia Cuoco, Paolo Barone, Jaime Kulisevsky, Angelo Antonini, Roberta Biundo

**Affiliations:** 1 Parkinson and Movement Disorders Unit, Study Center for Neurodegeneration (CESNE), Department of Neuroscience, University of Padua, Via Giustiniani 2, 35128 Padua, Italy; 2 Padua Neuroscience Center (PNC), University of Padua, 35131 Padua, Italy; 3 Parkinson’s Disease and Movement Disorders Unit, Center for Rare Neurological Diseases (ERN-RND), Department of Neurosciences, University of Padova, Padua, Italy; 4 IRCCS San Camillo Hospital, Via Alberoni 70, 30126 Venice, Italy; 5 Research Institute On Health Sciences (IUNICS), Balearic Islands Health Research Institute (IdISBa), University of the Balearic Islands, Balearic Islands, Spain; 6 Movement Disorders Rehabilitation Department, Moriggia-Pelascini Hospital, Via Pelascini 3, Gravedona Ed Uniti, Gravedona, Italy; 7 Parkinson Institute Milan, ASST G. Pini-CTO, Via Bignami 1, 20126 Milan, Italy; 8 Department of Neurology, University Hospital of Würzburg, Julius Maximilian University of Würzburg, Josef-Schneider-Straße 11, 97080 Würzburg, Germany; 9 Department of Psychology, University of Campania “Luigi Vanvitelli”, Viale Ellittico, 31, Caserta, Italy; 10 Department of Medicine, Surgery and Dentistry “Scuola Medica Salernitana”, Neuroscience Section, University of Salerno, Baronissi, Salerno, Italy; 11 Movement Disorders Unit, Sant Pau Hospital, Hospital Sant Pau, C/ Mas Casanovas 90, 08041 Barcelona, Spain; 12 Universitat Autònoma de Barcelona, Barcelona, Spain; 13 CIBERNED (Network Centre for Neurodegenerative Diseases), Madrid, Spain; 14 Department of General Psychology, University of Padua, Padua, Italy

**Keywords:** Parkinson’s disease, Parkinson’s disease mild cognitive impairment, Parkinson’s disease dementia, PD-CFRS

## Abstract

A key distinguishing factor between mild cognitive impairment (MCI) and dementia in Parkinson’s disease (PD) lies in the notable decrease in functioning due to cognitive impairment. The Parkinson’s Disease-Cognitive Functional Rating Scale (PD-CRFS) was developed to assess functional limitations caused by cognitive impairment, while reducing the influence of motor impairment. The aim of this multicenter study was to (i) validate the Italian version of the PD-CFRS in PD, (ii) determine optimal cut-off scores for detecting MCI and dementia in PD, (iii) compare its performances with the most established functional assessment tool (IADL). Six hundred and sixty nine PD participants were recruited from 4 Italian Movement Disorders centers (Venice, Milan, Gravedona, and Salerno). They underwent Level-II cognitive evaluation, which resulted in 282 PD-NC, 310 PD-MCI, and 77 PDD. The PD-CFRS’s psychometric and clinimetric properties, applicability, and responsiveness were analyzed. The PD-CFRS showed high acceptability. Floor and ceiling effects were acceptable. It also displayed strong internal consistency (Cronbach’s α = 0.738), and test–retest reliability (ICC = .854). The PD-CFRS demonstrated higher coefficient of variation to detect dysfunction in PD-MCI patients in comparison to the IADL scale (PD-CFRS 96% vs IADL 22.5%). Convergent validity with the IADL was r = − 0.638 and − 0.527 in males and females, respectively. PD-CFRS total score negatively correlated with global cognition (MoCA corrected score r = − 0.61; p < 0.001). A cut-off score > 6.5 identified PDD with a sensitivity of 90% and specificity of 88% (AUC = .959). A cut-off value of > 1 detected PD-MCI with a sensitivity of 68% and specificity of 69% (AUC = .695). The Italian version of the PD-CFRS demonstrated to be an easy, valid and reliable tool that properly captures functional impairment due to cognitive decline in PD. It also proved to be particularly effective in the advanced stages of PD, and would be a useful support for the diagnosis of PD-MCI and PDD.

**Supplementary Information:**

The online version contains supplementary material available at 10.1007/s00702-024-02746-6.

## Introduction

Cognitive impairment is possibly the most important and invalidating poorly levodopa-responsive symptoms in Parkinson’s disease (PD) (Antonini et al. 2023). Its presence negatively affects patients’ life expectancy and is associated with poorer QoL (Rosenthal et al. 2010). The full spectrum of cognitive deterioration ranges from subtle cognitive decline to mild cognitive impairment (PD-MCI) and dementia (PDD), with great heterogeneity in its presentation, severity, and rate of progression (Aarsland et al. 2021). According to a systematic review and meta-analysis of 39 studies representing 4011 patients with PD, 20% of people with PD-MCI developed PDD within 3 years (Saredakis et al. 2019). Although PD-MCI may not always progress to PDD, MCI constitutes a potential harbinger of conversion to dementia (Pedersen et al. 2013, 2017; Wood et al. 2016; Hoogland et al. 2017) occurring in up to 80% of patients with longer PD durations, especially after 15 to 20 years (Hely et al. 2008). The presence of dementia leads to a significant decline in Health-Related Quality of Life (HR-QoL), greater neuropsychiatric and motor alterations, increased caregiver burden and earlier nursing home placement (Lawson et al. 2017; Fan et al. 2020). Therefore, early detection and characterization of cognitive impairment is critically relevant for predicting future cognitive decline and providing adequate clinical care.

Approximately 25.8% of non-demented patients with PD have MCI (Aarsland et al. 2021). However, the reported estimated prevalence ranges between 9 and 65% of Parkinson’s disease cohorts, demonstrating the challenge in defining and diagnosing MCI. Impaired functional independence resulting from cognitive decline is a critical criterion to discriminate PD-MCI from PDD (Litvan et al. 2012). However, concurrent motor complications inherent to the disease and overlapping features with MCI-multidomain subtype makes it challenging to differentiate the contributions of cognitive or motor influences to daily functional tasks in PD. In that sense, defining the precise impact of cognitive impairment while reducing the effect of disease's motor symptoms is somewhat complex (Aarsland et al. 2017). This estimation is further complicated by the dearth of brief, reliable tools to specifically quantify functional changes related to cognitive impairment on patient’s daily life activities.

The Activities of Daily Living (ADL) scale (Katz et al. 1970) and the Instrumental Activities of Daily Living (IADL) scale (Lawton et al. 1969) are two widely used assessment tools. However, these instruments lack to measure the effect of cognitive dysfunctions on functional impairment as both instruments were not developed for disorders which include predominant motor symptoms. The Parkinson’s Disease-Cognitive Functional Rating Scale (PD-CRFS), designed by Kulisevsky et al. (2013) is a PD-specific instrument designed to explore the full spectrum of functional decline due to cognitive deterioration, minimizing the motor impact of the disease. Its strong psychometric properties also extend to different conditions and levels of cognitive decline in distinct clinical populations such as MCI and Alzheimer’s disease (AD) (Ruzafa-Valiente et al. 2016).

The PD-CRFS was developed and validated (Kulisevsky et al. 2013), but it has not been validated in the Italian population. Based on these considerations, the purpose of the present multicenter study was first, to validate the Italian version of PD-CRFS in a large PD cohort, representative of the Italian population, and second, to determine optimal cut-off scores for detecting MCI and dementia in PD. Furthermore, a comparative analysis with the most established functional assessment tool (IADL) has been included, to test PD-CFRS properties in assessing cognitive decline.

## Methods

### Participants

This study was conducted in four Italian Movement Disorders centers -Venice, Milan, Gravedona, and Salerno—representative of the Italian population present in rural, suburban areas and in city centers in northern and southern Italy. PD patients were consecutively enrolled if they (1) were native Italian-speakers, (2) had provided written and signed informed consent form (3) met Brain Bank diagnostic criteria for probable PD (Postuma et al. 2018) from de novo to severe form (4) had the diagnosis confirmed by a DAT-Scan (5) were accompanied by a native Italian-speaking caregiver providing daily supervision and assistance to the patients with PD. Patients with a history of deep-brain stimulation surgery at the time of the assessment, CT or MRI abnormalities, head injury, current or history of alcohol or drug abuse, psychiatric disorders, stroke or other concomitant neurological illness or severe sensorial deficits detected on a semi-structured clinical interview were excluded.

**Fig. 1:**
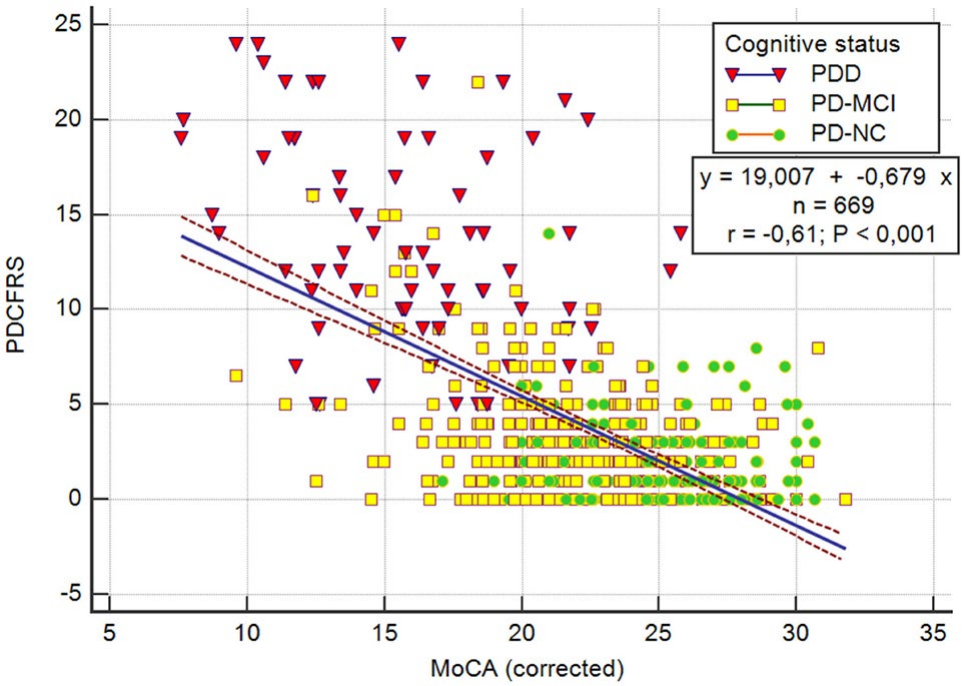
Correlation between the global cognition measure Montreal Cognitive Assessment (MoCA) and the Italian version of Parkinson’s Disease Cognitive Functional Rating Scale (PD-CFRS) in our PD cohort

A total of 669 idiopathic PD patients and 119 healthy controls (HCs) matched for age, age range, education and sex were enrolled as a part of the ongoing project “Validation of Mild Cognitive Impairment criteria in Italian Parkinson’s disease patients” (GR-2016-02361986) (see Figs. 1, 2 from Supplementary material for further details). Based on their cognitive profile, patients were categorized as follows: 282 PD with normal cognition (PD-NC), 310 PD-MCI and 77 PDD.

**Fig. 2:**
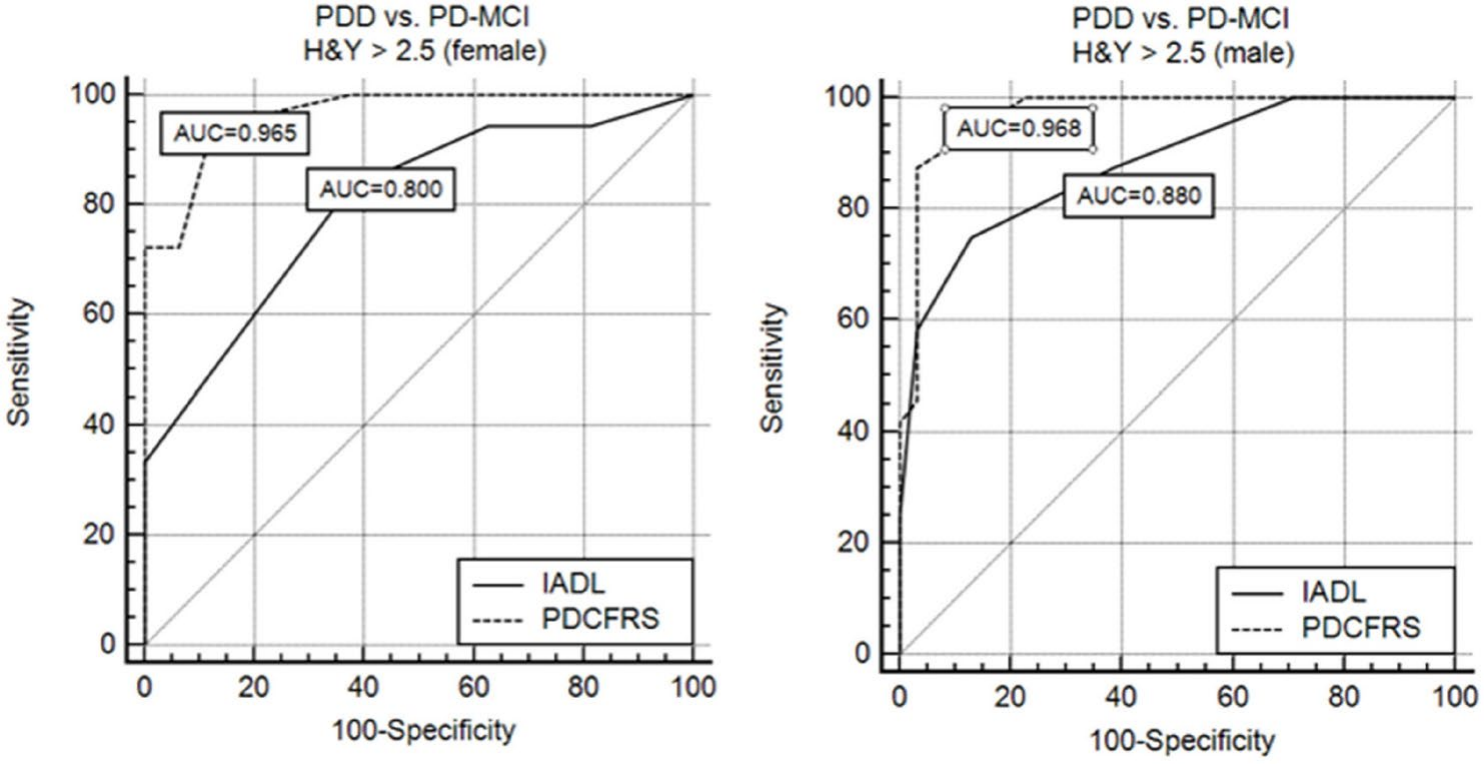
Cognitive discriminative power of the Italian version of Parkinson’s Disease Cognitive Functional Rating Scale (PD-CFRS) and IADL in presence of severe motor deficit in PD population

The present study was approved by the Venice San Camillo Research Ethics Committee, in Venice, Italy. Written informed consent was obtained from all study subjects after full explanation of the procedure involved. The research was completed in accordance with the Declaration of Helsinki.

### Neurological and neuropsychological assessment

All patients underwent an extensive motor, behavioral, and neuropsychological evaluation. All patient were advised to adhere to their usual schedule of PD medications for their study visit, ensuring they were assessed in their “on” state. All assessments were carried out in two consecutive visits with a maximum interval of two weeks. Demographic and clinical variables included age, years of education, sex, age of onset, disease duration, levodopa equivalent doses (LEDD), dopamine agonist equivalent daily dose (DAED), and motor severity assessed by the Movement Disorder Society Unified Parkinson's Disease Rating Scale motor score (MDS-UPDRS III) and Hoehn & Yahr Staging (H&Y). LEDD and DAED were calculated according to Tomlinson et al. (2010). The Italian version of the PD-CFRS was administered by a trained neuropsychologist to all PD patients with the presence of a caregiver, within one week from visit and along with the routinary daily functioning scales (ADL/IADL scales). The average time needed to complete the PD-CFRS was 15 ± 2 min and the free version of the scale is available at the link https://www.movementscales.com/formulario-func-italian. For PD-CFRS administration and scoring details see the original article (Kulisevsky et al. 2013).

Cognitive assessment was administered by neuropsychologists with experience in movement disorders to determine patients' cognitive status according to the MDS Task Force Level II diagnostic criteria for PDD (Emre et al. 2007) and PD-MCI (Dubois et al. 2007; Litvan et al. 2012). For further details on the cognitive tests adopted see Fiorenzato et al. (2019).

Patients without cognitive deficits were defined as cognitively normal (PD-NC). Cognitive impairment was defined as performance of ≤ -1.5 standard deviations (SDs) below age and education-matched norms. PD-MCI was differentiated from PDD based on the clinical judgment derived from extensive neurological and clinical evaluation including presence of hallucinations, interview with caregivers and consensus between professional figures. Further, the presence of traits of depression, anxiety, apathy, impulsiveness and QoL were assessed using the Beck Depression scale (BDI-II) (Beck et al. 1996), State-Trait Anxiety Inventory (STAI-I and II) (Pedrabissi and Santinello 1989), Starkstein’s Apathy Scale (AS) (Starkstein et al. 1992) and Barrat Impulsiveness Scale (BIS-11) (Fossati et al. 2001) and the 8-item version of Parkinson's disease quality of life (PDQ-8) (Yamanishi et al. 2013), respectively.

At the second visit, PD-CFRS was re-administrated by the same neuropsychologist at two-week intervals in a subsample of 84 PD patients.

### Statistical analyses

Descriptive statistics were calculated for demographic and clinical variables. Continuous and discrete clinical characteristics were compared with Welch test and chi squared. A p < 0.05 Bonferroni corrected (p = 0.0022 adjusted p value) was set for significance.

After checking for missing data (acceptable < 5%), the following psychometric attributes were explored for the PD-CFRS: acceptability, internal consistency, convergent construct validity, reliability, and discriminant validity. For acceptability, a 95% value of computable data for each PD-CFRS item was considered appropriate. 15% was accepted as maximum value for floor and ceiling effect (i.e., lowest and highest possible scores, respectively). Internal consistency (i.e., the degree to which the set of items in the scale covaries in relation to their sum score) was assessed with Bayesian Cronbach's α coefficient (acceptable value: ≥ 0.70). Moreover, Item drop analysis was used to evaluate Bayesian Individual Item Reliability Statistics and possible improvement in consistency after each item removal. Coefficient of variation (CV) was calculated for PD-CFRS and IADL and a discriminative validity study (t-test) was used to determine their ability to differentiate between PD cognitive groups. Since IADL refers to different abilities in male and female, CV calculation was run grouped by sex. Convergent validity was assessed using Spearman Correlation to explore relations between PD-CFRS and MMSE, MoCA, and each MDS-UPDRS subscale. Moreover, the functional impact due to cognitive impairment was analyzed by the correlation coefficient in the univariate linear regression between PD-CFRS with MoCA within the whole cognitive spectrum. Pearson partial correlation was used to assess PD-CFRS convergent validity with MMSE and MoCA, using as covariates age of onset, education, disease duration, MDS-UPDRS III, BDI-II, AS, BIS-11, STAI Y1 and Y2. Concurrent validity was assessed using Spearman Correlation and between PD-CFRS and ADL, IADL. A single rater test–retest Interclass correlation ICC3,1 was performed to examine participant’s performance reliability at 2-week evaluation. In addition, the Bland Altman analysis was used to evaluate the agreement between each test–retest. Following the procedures of the original validation of the scale, discriminant validity of the PD-CFRS was evaluate through binary logistic regression (stepwise; conditional) analysis including PD-CFRS, MMSE-corrected score, MoCA-corrected score, disease duration at visit as independent variables (enter p < 0.01, remove p > 0.1). Sensitivity, specificity, and receiver operating curves (ROCs) with the area under the curve (AUC) analyses were conducted to establish cut-off scores to discriminate among cognitive statuses. The discriminative power of MMSE, MoCA and PD-CFRS was also compared. The screening cut-off point was defined as the value achieving > 80% sensitivity and Negative Predictive Value (NPV). The diagnostic cut-off point was defined as the value achieving > 80% specificity and Positive Predictive Value (PPV). Finally, we also compared the accuracy of the PD-CFRS with respect to the IADL scale. ROCs curves were generated to evaluate the discriminative power of both instruments as screening tools for PDD in presence of severe motor deficit (defined as H&Y > 2.5) and considering possible gender biases. IBM-SPSS version 25.0 and JASP 0.16.4 software were used for these analyses; p < 0.05 was considered statistically significant.

## Results

### Demographic and clinical data

The sociodemographic and clinical characteristics of the whole sample (PD = 669 and HC = 119) are summarized in Table 1. HC and PD differed significantly in PD-CFRS scores [score range = 0–3, mean ± SD = 0.56 ± 0.86 vs. scores range = 0–19, mean ± SD = 3.8 ± 4.8 respectively (p < 0.0001)]; IADL [mean ± SD = 6.3 ± 1.5 vs. mean ± SD = 5.13 ± 1.8, respectively (p < 0.0001)]; ADL [mean ± SD = 6 ± 0 vs. mean ± SD = 5,4 ± 1.3 respectively (p < 0.0001)]; cognitive states [NC: 66,4% vs. 42,2%; MCI: 33,6% vs. 46,3%; dementia: 0% vs. 11,5%, respectively (p < 0.0001)]; MMSE- corrected score [mean ± SD = 27.3 ± 2.5, vs. mean ± SD = 25.9 ± 3.6, respectively, (p < 0.0001)] MoCA- corrected score [mean ± SD = 24.6 ± 2.9 vs. mean ± SD = 22.6 ± 4.3, p < 0.0001)]; clinically relevant depressive traits (BDI-II > 14) (moderate to severe depression 2.5% vs 25,5%, p < 0.0001); and clinically relevant anxiety traits (STAI-Y2) (17% vs. 42,4%, p < 0.002).

**Table 1:** Demographic and clinical characteristics of the PD and HC samples

	HC N = 119			PD N = 669			Welch test/chi squared
	Mean	SD	2.5–97.5 P	Mean	SD	2.5–97.5 P	Two-taled probability (df = 788)
Age at visit	67.706	8.937	50.9–84.0	67.138	10.285	46.0–85.0	P = 0.4071
Sex (% Male)		57.1%			65.70%		P = 0.0895
Age at onset				56.967	11.289	35.3–76.0	/
Disease duration				10.077	5.99	1.0–23.7	/
Education	11.370	4.353	5.0–19.0	11.383	4.53	5.0–18.0	P = 0.2713
Education < 8		18.5%			19.8%		P = 0.832
LEED				701.430	405.78	100.4–1708.3	/
DAED				146.46	108.552	0.0–480.0	/
MDS-UPDRS-I				11.239	6.57	1.05–26.9	/
MDS-UPDRS-II				13.000	7.49	1.0–32.4	/
MDS-UPDRS-III				26.455	15.28	6.0–63.9	/
MDS-UPDRS-IV				3.68	3.788	0.0–12.0	/
H&Y > 2.5					33.0%		/
ADL	6	0	6.0–6.0	5.368	1.239	1.0–6.0	P < 0.0001^**&**^
IADL	6.277	1.48	5.0–8.0	5.127	1.786	1.0–8.0	P < 0.0001^**&**^
PD CFRS	0.563	0.86	0.0–3.0	3.735	4.840	0.0–19.0	P < 0.0001^**&**^
BDI-II > 14		2.5%			25.5%		P < 0.0001^**&**^
APATHY scale > 14		10.6%			29.0%		P = 0.0156
STAI-Y1 > 40		14.9%			28.2%		P = 0.0905
STAI-Y2 > 40		17%			42.4%		P = 0.0020^**&**^
MMSE-Corrected score	27.366	2.470	21.5–30.0	25.967	3.55	16.4–30.0	P < 0.0001^**&**^
MoCA -Corrected score	24.6	2.85	18.4–29.6	22.607	4.30	12.34–29.3	P < 0.0001^**&**^
Cognitive State NC/MCI/Dementia %	66.4%/33.6%/0%			42.2%/46.3%/11.5%			P < 0.0001^**&**^

^&^ Significant after Bonferroni corrected threshold (p < 0.05) = 0.0022

### Data quality and test acceptability

All data were computable and there was no missing value for any item of the PD-CFRS. The overall PD sample showed significantly lower frequency of floor effect compared with the overall HC group (mean: 62.9% vs. 28.3%, p < 0.0001). In particular, non-demented PD patients displayed significantly lower percentage of floor effect in PD-CFRS (score = 0) rather than in IADL score (score = 8) (28% vs. 72%, p < 0.0001), with only 38.4% in PD-NC. This was confirmed by large differences in the coefficients of variation (CV) indicating that the PD-CFRS gathered a broader range of data with respect to IADL scale, reported separately for females and males respectively (129% vs. 14–10% CV in PD-NC and 96% vs. 26–19% CV in PD-MCI).

Regarding PD-CFRS (score = 24) ceiling effect in PDD, it was present in only 5% of the cases, similarly or below the frequency observed in IADL (7–12%, females and males) (see Table 2).

**Table 2:** Acceptability of the Italian version of Parkinson’s Disease Cognitive Functional Rating Scale (PD-CFRS)

	PDCFRS		IADL female		IADL male	
	HC	PD	HC	PD	HC	PD
Floor effect (PDCFRS = 0 |IADL = 8 | IADL = 5)						
NC	65.0%	38.4%	100%	71.1%	100%	90.5%
MCI	60.8%	18.2%	96.0%	47.1%	100%	67.7%
Dementia	/	0	/	0%	/	0%
Ceiling effect (PDCFRS = 24 | IADL = 0						
NC	0%	0%	0%	0%	0%	0%
MCI	0%	0%	0%	0%	0%	0%
Dementia	/	4.90%	/	7.7%	/	12.2%
% of values computable						
100%	100%	100%	100%	100%	100%	100%
% missing value						
0%	0%	0%	0%	0%	0%	0%
Coefficent of variation						
NC	141%	129%	0%	14%	0%	10%
MCI	165%	96%	2.5%	26%	0%	19%
Dementia	/	38%	/	55%	/	61%
Mean (SD)						
NC	0.52(0.73)	1.32(1.63)	8 (0)	7.46 (1.04)	5 (0)	4.86(0.48)
MCI	0.65(1.08)	3.25(3.24)	7.96(0.2)	6.59(1.69)	5 (0)	4.49(0.85)
Dementia	/	14.0(5.37)	/	3.42(1.90)	/	2.00(1.22)

### Internal consistency and reliability

Internal consistency results are shown in Supplementary Fig. 4. The PD-CFRS demonstrated strong internal consistency (Bayesian Cronbach’s α = 0.738, CI: 95%, 0.604–0.849). Average inter-item correlation was 0.289 (CI: 95%, 0.173–0.398). No item improved Bayesian Cronbach’s α if removed.

Same rater Test –retest analysis showed high interclass correlation (ICC3,1 = 0.854; CI 95%, 0.783–0.903).

Bland Altman plot evidenced that test–retest mean limits range from + 5.125 (CI 95%, 4.2–6.0) to – 4.268 (CI 95%, − 5.2 − 3.4) (see Supplementary-Fig. 5).

### Convergent and concurrent construct validity

The convergent validity analysis between the PD-CFRS and the other measures considered are displayed in Supplementary Table 1. Spearman rank analysis showed a significant correlation with demographic data. In particular, correlations between PD-CFRS scores and age, age at onset, low education and DEAD were revealed (p < 0.0001). No significant correlation was found with LEDD.

Regarding motor variables, PD-CFRS score worsened with higher motor deficits (UPDRS –III, H&Y) (p < 0.0001), but no significant correlation was found with the presence of dyskinesia and fluctuations as measured by MDS-UPDRS-IV.

Overall, PD-CFRS score was strongly correlated with the presence of non-motor symptoms and global cognitive deficits, as measured by the MDS-UPDRS-I, MoCA and MMSE (p < 0.0001).

From a behavioral standpoint, PD-CFRS score was associated with the presence of clinically relevant behavioral traits, evidenced by the correlation with MDS-UPDRS –II, STAI Y1-Y2, AS, BDI-II and BIS-11(p < 0.0001).

The impact of cognitive decline on functional impairment was examined by calculating the correlation coefficient in the univariate linear regression for the association between PD-CFRS with MoCA within the continuum from PD-NC to PDD. A higher and significant overall correlation (r = -0.61, p < 0.001) was found in each cognitive status (See Fig. 1).

Pearson partial correlations revealed a significant association between PD-CFRS and MoCA (r = -0.271, p < 0.0002) and MMSE (r = -0.217, p < 0.0029), respectively.

Finally, an optimal concurrent validity was found with the IADL scale, both in males and females (p < 0.0001).

### Discriminant validity

Stepwise logistic regression showed that PD-CFRS outperformed other widely used global scale as screening tool for discriminating II-level PD-MCI from PD-NC (OR: 1.3, CI 95%, 1.18–1.44) and PDD from PD-MCI (OR: 1.46, CI 95%, 1.31–1.62).

Discriminant ROC analysis (AUC = 0.695 [95% CI 0.656–0,731]) showed that PD-CFRS screening cut-off score for detecting functional impairment in PD-MCI was > 0 [(SEN = 0.80; SPE = 0.39; PPV = 0.5870; NPV = 0.8067)] and the optimal cut-off was > 1 [(SEN = 0.68; SPE = 0.69; PPV = 0.701; NPV = 0.6783)]. An optimal cut-off score of > 6.5 (SEN = 0.90; SPE = 0.88; PPV = 0.64; NPV = 0.98) was found to be optimal for detecting PDD (AUC = 0.959 [95% CI 0.935–0.976]) (see Table 3 and Supplementary Fig. 6).

**Table 3:** Accuracy measures of the Italian version of Parkinson’s Disease Cognitive Functional Rating Scale (PD-CFRS) for detecting functional impairment in PD

Cut-off	Diagnostic cut-off	Optimal cut-off	Screening cut-off	Sensitivity	Specificity	PPV %	NPV %	AUC (CI 95% CI)
PD-MCI vs. PD-NC	> 4			0.25	0.94	0.82	0.54	0.695 (0.656–0.731)
		> 1		0.68	0.69	0.70	0.67	
			> 0	0.80	0.39	0.58	0.80	
PDD vs. PD-MCI	> 9			0.78	0.96	0.81	0.95	0.959 (0.935–0.976)
		> 6.5		0.90	0.88	0.64	0.98	
			> 5	0.99	0.84	0.58	0.98	

*MCI* Mild cognitive impairment, *NC* Normal Cognition, *PDD* PD Dementia, *PPV* Positive Predictive Value, *NPV* Negative Predictive Value, *AUC* Area Under the Curve

Test value at specificity and PPV of 80%; Test value at sensitivity and NPV of 80%

Finally, ROC analyses showed that IADL and PD-CFRS performed similarly when there were no severe motor deficits (H&Y ≤ 2.5). On the other hand, PD-CFRS significantly outperformed IADL in capturing cognitive dysfunctions in PD in presence of concurrent motor deficits that might have a relevant impact on the patient daily life (H&Y > 2.5), both in females (AUC = 0.965 [95% CI: 0.839–0.999] vs. 0.800 [95% CI 0.628–0.917], p < 0.0235) and in males (AUC = 0.968 [95% CI 0.882–0.997] vs. 0.880 [95% CI 0.765–0.952], p < 0.062) (See Fig. 2).

## Discussion

This is the first multicentric study to validate the Italian version of PD-CFRS in a large cohort of patients with PD, covering the full spectrum of cognitive impairment and classified according to the II Level diagnostic criteria proposed by the MDS Task Force for PD-MCI and PDD.

Our data confirm that compared to the most commonly used scale (IADL), PD-CFRS is a valid and more reliable instrument that properly captures functional impairment due to cognitive decline in PD (Kulisevsky et al. 2013) even in the presence of a severe motor profile (H&Y > 2.5).

The PD-CFRS scale displays similar psychometric properties to those of the original study (Kulisevsky et al. 2013). Importantly, our work extends previous findings supporting evidence on the ability to capture cognitive-related functional impairment regardless of gender and motor severity. In contrast to the most widely used functional activity measurement instruments (e.g., IADL; ADL), the PD-CFRS allows to minimize motor biases while capturing the functional impact of cognitive impairment in PD and thus, to adequately capture the clinical significance of cognitive changes along the disease course (Choi et al. 2019). Likewise with previous evidence (Pagonabarraga et al. 2008), the PD-CFRS showed high acceptability since data were all computable without missing values. The acceptability of the Italian version was confirmed by the low frequency of the ceiling effect in PDD (5%). Similar to the Spanish validation, 18% of PD-MCI patients did not exhibit functional impairment on PD-CFRS (floor effect). However, comparing the CV between PD-CFRS and IADL (96% vs. 20%), a significant improvement in the data gathered can be observed. The Italian version of the PD-CFRS presents a high, acceptable internal consistency (Cronbach’s-α = 0.738; corrected item-total correlation = 0.289), which is close to the values shown in the Spanish version (Cronbach’s-α = 0.797). Our results revealed a significant inter-item correlation, reinforcing the excellent reliability of this scale.

With regard to convergent validity, PD-CFRS strongly correlated with the global cognitive scales, MMSE and MoCA, the most used scales for the assessment of cognition in PD (Biundo et al. 2013, 2014). The strength of the association survived after adjusting for possibly influencing factors such as age, education, disease duration, motor status and behavioral traits, demonstrating that PD-CFRS is reliable in measuring the cognitive impact associated with functional impairment. Furthermore, in assessing concurrent validity, we compared the PD-CFRS scale to the gold standard, the IADL scale, and the correlation found underscores the robustness of its construct validity.

Additionally, we demonstrated within our cohort the superiority of the PD-CFRS over the IADL in distinguishing between PDD and PD-MCI, in the presence of high motor disability (H&Y > 2.5). Whereas both scales demonstrated to be equivalent in terms of discriminative power in the case of low motor impairment (H&Y < 2.5), when the latter increases, PD-CFRS maintains high discriminative power, proving to be particularly effective in the advanced stages of PD. In fact, the most used clinical scales that assess the ability to perform functional activities of daily living (ADL and IADL) are significantly distorted by motor impairment, requiring additional clinical assessment to account for cognitive interference.

The test–retest analysis (Bland Altman plot) provided the Minimum Detectable Change (MDC) over a 2-week period, highlighting that the changes between 5.125 (worsening of performance) and – 4.198 (amelioration of performance) score points are not statistically significant.

Timely identification of functional impairment due to cognitive decline is crucial for ameliorating accuracy in PD-MCI detection, as well as for tracking disease progression and planning the most appropriate interventions.

The PD-CFRS also demonstrated excellent discriminative accuracy in differentiating PD dementia from PD-MCI. An optimal cut-off score of > 6.5 identified PDD with a sensitivity of 90% and specificity of 88%, with an AUC of 0.959. Discrimination between PD-NC and PD-MCI was also possible, with an AUC of 0.695. A cut-off value of > 1 detected PD-MCI with a sensitivity of 80% and specificity of 39%.

This reduced specificity in discriminating the PD-NC from very mild PD-MCI may be attributed to the II level cognitive evaluation used in the present study. In fact, it has been reported that using a restricted number of tests or cognitive domains for the evaluation, as well as employing various cut-off scores below the mean of age-adjusted normative data (-1SD, -1.5 SD, -2 SD) poses a challenge for PD-MCI discrimination from PD-NC (Dalrymple-Alford et al. 2011; Cammisuli et al. 2019). Our use of a comprehensive neuropsychological testing and adherence to MDS Task Force Level II diagnostic criteria could have led to a more accurate screening of PD-MCI, minimized the risk of false positives and revealed multiple cognitive deficits that might otherwise be undetected. Recent findings suggest that the added value of level II PD-MCI lies in its higher sensitivity allowing for the inclusion of patients with varying degrees of cognitive severity, ranging from mild to severe, in the PD-MCI group (Hoogland et al. 2017).

Our cut-off scores slightly differed from those established by Kulisevsky et al. (2013). According to the original study (Kulisevsky et al. 2013), scores of ≥ 3 and ≥ 6 were identified as optimal for detecting PD-MCI and PDD, respectively. The different cognitive evaluation level and diagnostic criteria adopted between the present work and the original validation study, could have influenced the determination of the clinical cut-offs. Furthermore, this difference may be attributed to the different administration modality: while in the Spanish version the test was completed by a knowledgeable informant, in our study it was administered by an expert neuropsychologist trained in movement disorders.

Previous research has explored the difference between self-reported and objectively measured performance, particularly in the context of MCI, where there is a tendency to overstate functional and cognitive abilities (Okonkwo et al. 2009). Indeed, several studies have offered additional evidence regarding the trustworthiness of caregivers' self-reports when compared to those of patients (Seltzer et al. 2001; Leritz et al. 2004). However, it's essential to recognize that a clinician's interpretation of a patient's responses may influence reporting differently and may better capture symptoms compared to information reported by the patients themselves.

Furthermore, it is noteworthy to mention that around 88% of male and 79% of female individuals with PD appoint an informal caregiver, and that individuals with higher levels of functioning may not yet need a caregiver (Prizer et al. 2020). Exploring an alternative administrative approach, which depends on the expertise of clinicians, might offer the potential for a more impartial assessment. This strategy could address the difficulty of gaining a comprehensive understanding of the disease when no caregiver is available. There are several limitations that should be acknowledged in the current study. Firstly, we did not explore variance or the concordance between patients and caregivers self-reported information. Secondly, we did not undertake a longitudinal analysis of the scale's responsiveness to assess its ability to detect changes in sensitivity over time. Thirdly, it is worth noting that various clinical and genetic phenotypes of PD may potentially exhibit diverse responses. Nevertheless, the extensive and multicenter sample size should have mitigated this limitation.

Another limitation could derive from the disparity in sample sizes between the healthy control group and the PD group (119 vs 669). While it's important to note that the PD-CFRS was not specifically designed and validated as a diagnostic or screening tool for PD, the uneven distribution of participants could introduce a potential bias and restrict the applicability of our findings for evaluating cognitive-related functional impairment in the broader elderly non-PD population. Furthermore, while achieving internal consistency consistent with the original Kulisevsky validation, the PD-CFRS underwent re-testing in a smaller subgroup of PD individuals (84 participants out of 669). Despite no significant differences in age, sex, motor severity (MDS-UPDRS-III), and global cognitive scales (MMSE, MoCA) compared to the entire sample, there is a potential risk of introducing a bias in generalizability.

Nevertheless, the current study presents notable strengths. The inclusion of a very large cohort of rigorously screened PD patients recruited from various specialized movement disorders centers across different geographic regions offers a representative cross-section of the Italian population, spanning from North to South Italy. Furthermore, all patients underwent thorough clinical and neuropsychological evaluations, enabling the application of a Level II cognitive diagnosis in accordance with the latest expert consensus recommendations (Aarsland et al. 2010; Litvan et al. 2011).

In line with prior research (Meredith A. Bock et al. 2023), our findings reinforce the notion that functional impairment associated with cognitive decline manifests prior to the onset of dementia.

In summary, considering the constraints on the time of healthcare professionals in clinical environments, the PD-CFRS is a concise and dependable tool that meets essential feasibility criteria for its application in clinical and research contexts across all stages, especially for PDD.

## Conclusion

In summary, this study offers insights into the psychometric properties of the PD-CFRS and identifies optimal thresholds that demonstrate its effectiveness as a screening tool for Italian patients with PD-MCI and dementia. Furthermore, when compared to the IADL scale, this instrument provides more reliable insights into cognitive status based on the extent of functional impairment. Specifically, it effectively addresses the challenge of distinguishing cognitive diagnoses in the presence of moderate to severe motor symptoms, which is often a complex issue. Our research findings affirm the reliability and validity of the PD-CFRS as a tool for assessing functional decline in individuals with Parkinson's disease across various stages of the condition. Consequently, it emerges as a practical and easily administered instrument suitable for both clinical and research purposes.

### Supplementary Information

Below is the link to the electronic supplementary material.

Supplementary File 1 (PDF 459 KB)

## Data Availability

The data that support the findings of this study are available upon reasonable request.
