# Supplementary material for: Validation of the Italian version of the Parkinson’s Disease- Cognitive Functional Rating Scale
Source: J Neural Transm (Vienna). 2024 Jan 27;131(4):305–14. doi: 10.1007/s00702-024-02746-6 (PMC11016123; doi:10.1007/s00702-024-02746-6)
Supplement: Supplementary file 1 — (PDF 459 KB) [file 702_2024_2746_MOESM1_ESM.pdf]

e-Figure 1. Study Flow diagram

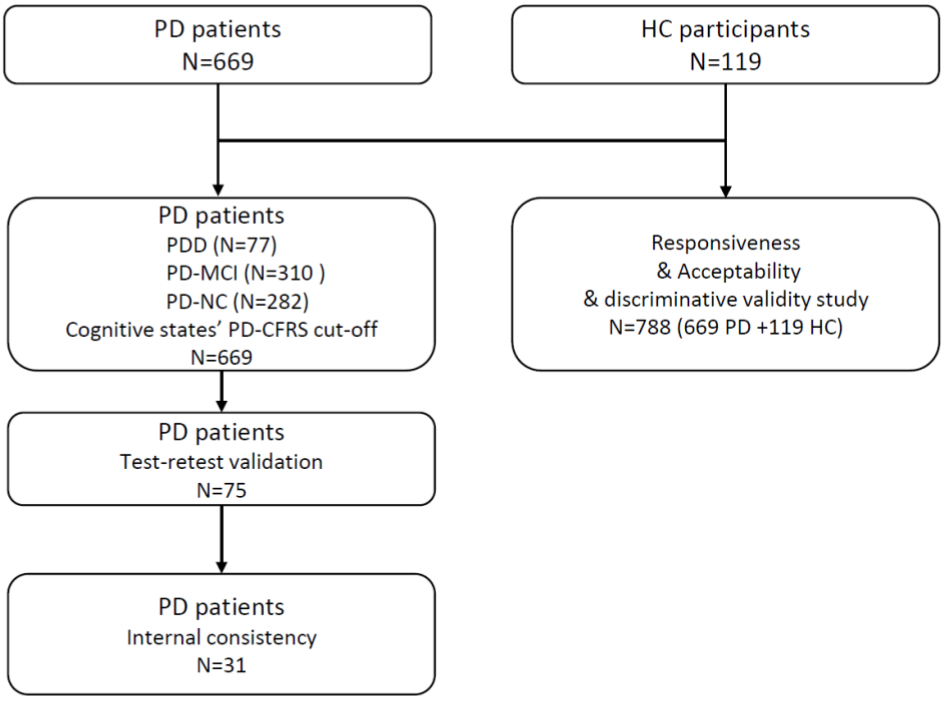

e-Figure 2. Participant recruitment distribution.

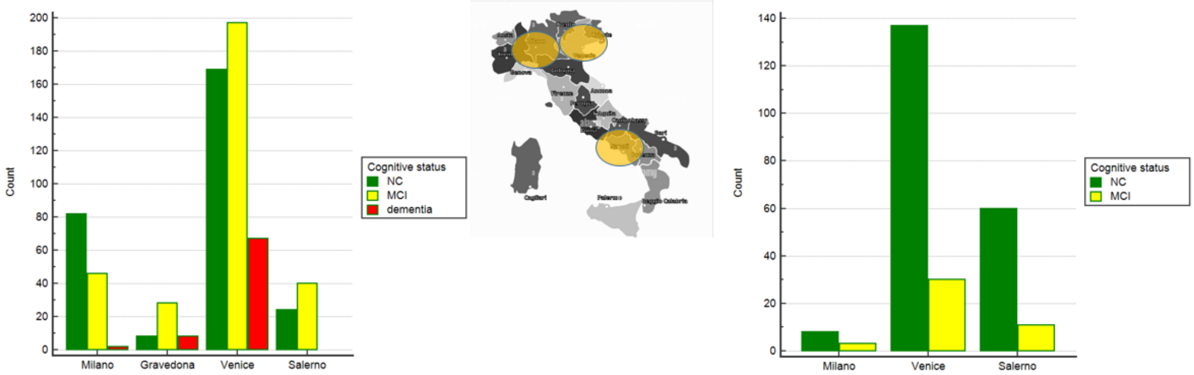

	Gravedona	Milano	Salerno	Venice	TOT
PD-NC	8	82	24	168	282 (41,9%)
PD-MCI	28	46	40	196	308 (45,9%)
PDD	8	2	0	67	82 (12.2%)
TOT	44	130	64	431	669

	Milano	Salerno	Venice	TOT
NC	5	11	63	40 (33,6%))
MCI	3	10	27	79 (66,4%)
TOT	11	71	90	119

e-Figure 3. Cognitive state distribution among disease duration and age in patients with Parkinson’s Disease

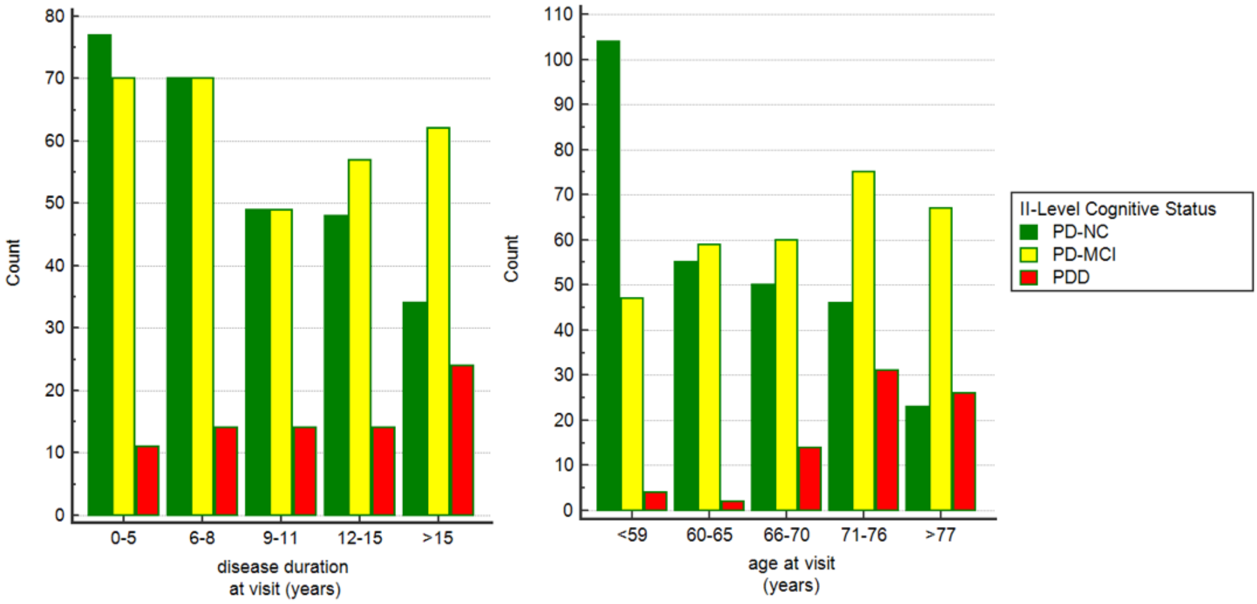

e-Figure 4. Italian version of Parkinson’s Disease Cognitive Functional Rating Scale. Cronbach's α If Item Dropped Posterior Plots.

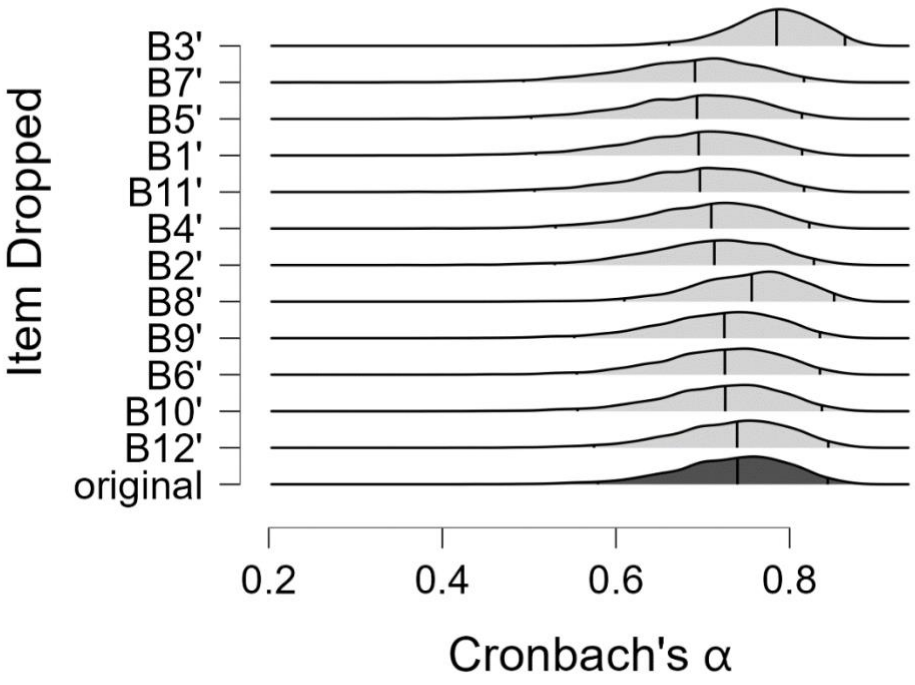

e-Figure 5. Italian version of Parkinson’s Disease Cognitive Functional Rating Scale test –retest reliability in PD population: Bland Altman Plot.

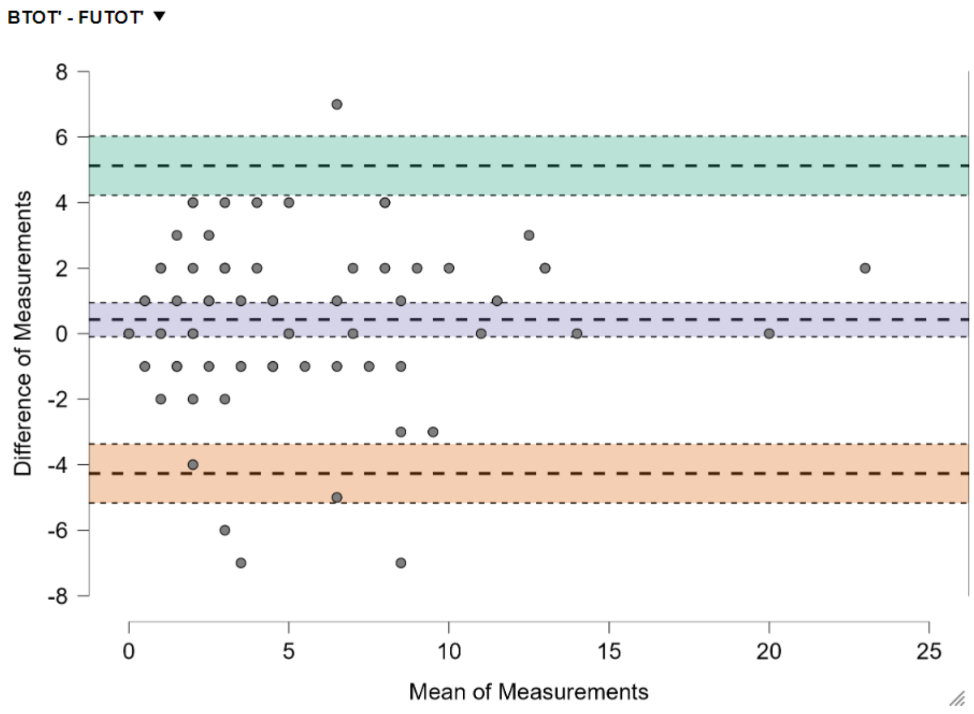

e Figure 6. Discriminant cut-off for detecting functional impairment associated to PDD.

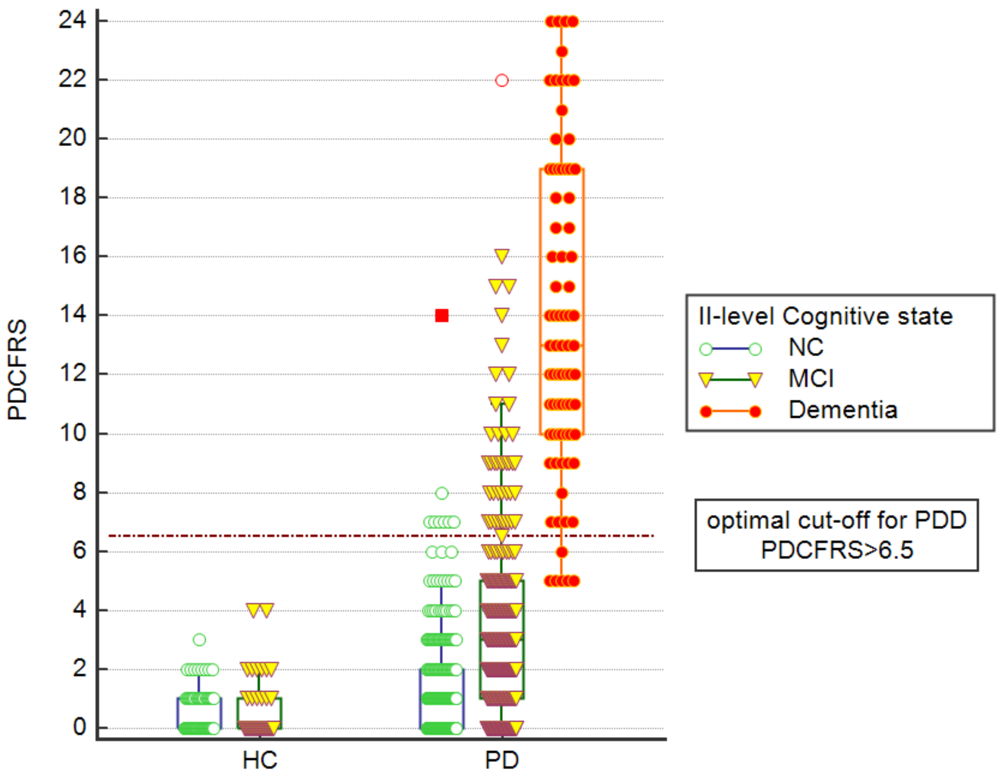

e-Table 1 Convergent and concurrent validity of the Italian version of Parkinson’s Disease Cognitive Functional Rating Scale

	Overall PD PDCFRS score N=669	
	Correlation Coefficient [r]	P value
Convergent Validity		
Age at visit	0,264	<0,0001
Age at motor onset	0,129	0,0009
Education	-0,212	<0,0001
LEED	-0,017	0,675
DAED	-0,136	0,0075
H&Y (ON)	0,465	<0,0001
MDS-UPDRS-I	0,43	<0,0001
MDS-UPDRS-II	0,483	<0,0001
MDS-UPDRS-III	0,463	<0,0001
MDS-UPDRS-IV	0,096	0,1019
MMSE (corrected score)	-0,442	<0,0001
MoCA (corrected score)	-0,474	<0,0001
STAI-Y1	0,283	<0,0001
STAI-Y2	0,333	<0,0001
APATHY scale	0,473	<0,0001
BDI-II	0,376	<0,0001
BIS-11	0,418	<0,0001
Concurrent Validity		
IADL (females)	-0,496	<0,0001
IADL (males)	-0,645	<0,0001
